# Concurrent Chemoradiotherapy Induces Body Composition Changes in Locally Advanced Head and Neck Squamous Cell Carcinoma: Comparison between Oral Cavity and Non-Oral Cavity Cancer

**DOI:** 10.3390/nu13092969

**Published:** 2021-08-26

**Authors:** Yu-Ching Lin, Hang Huong Ling, Pei-Hung Chang, Yi-Ping Pan, Cheng-Hsu Wang, Wen-Chi Chou, Fang-Ping Chen, Kun-Yun Yeh

**Affiliations:** 1 Department of Medical Imaging and Intervention, Chang Gung Memorial Hospital, College of Medicine, Keelung & Chang Gung University, Taoyuan 333007, Taiwan; yuching1221@gmail.com; 2 Osteoporosis Prevention and Treatment Center, Chang Gung Memorial Hospital, Keelung 20401, Taiwan; fangping@cgmh.org.tw; 3 Division of Hemato-Oncology, Department of Internal Medicine, Chang Gung Memorial Hospital, College of Medicine, Keelung & Chang Gung University, Taoyuan 333007, Taiwan; xianfang87@gmail.com (H.H.L.); ph555changg@gmail.com (P.-H.C.); chw0098@gmail.com (C.-H.W.); 4 Department of Nutrition, Chang Gung Memorial Hospital, Keelung 20401, Taiwan; pyngpyng@gmail.com; 5 Division of Hemato-Oncology, Department of Internal Medicine, Chang Gung Memorial Hospital, College of Medicine, Linkou & Chang Gung University, Taoyuan 333007, Taiwan; wenchi3992@yahoo.com; 6 Department of Obstetrics and Gynecology, Chang Gung Memorial Hospital, Keelung 20401, Taiwan; 7 Healthy Aging Research Center, College of Medicine, Chang Gung University, Taoyuan 333007, Taiwan

**Keywords:** head and neck cancer, concurrent chemoradiotherapy, lean body mass, total fat mass, bone mineral content, DXA

## Abstract

Few prospective cohort trials have evaluated the difference in treatment-interval total body composition (TBC) changes assessed by dual-energy X-ray absorptiometry (DXA) between two patient subgroups with locally advanced head and neck squamous cell carcinoma (LAHNSCC) receiving concurrent chemoradiotherapy (CCRT): oral cavity cancer with adjuvant CCRT (OCC) and non-oral cavity with primary CCRT (NOCC). This study prospectively recruited patients with LAHNSCC. Clinicopathological variables, blood nutritional/inflammatory markers, CCRT-related factors, and TBC data assessed by DXA before and after treatment were collected. Multivariate linear regression analysis identified the factors associated with treatment-interval changes in body composition parameters, including lean body mass (LBM), total fat mass (TFM), and bone mineral content (BMC). A total of 127 patients (OCC (*n* = 69) and NOCC (*n* = 58)) were eligible. Body composition parameters were progressively lost during CCRT in both subgroups. Extremities lost more muscle mass than the trunk for LBM, whereas the trunk lost more fat mass than the extremities for TFM. BMC loss preferentially occurred in the trunk region. Different factors were independently correlated with the interval changes of each body composition parameter for both OCC and NOCC subgroups, particularly mean daily calorie intake for LBM and TFM loss, and total lymphocyte count for BMC loss. In conclusion, treatment-interval TBC changes and related contributing factors differ between the OCC and NOCC subgroups.

## 1. Introduction

Head and neck squamous cell carcinoma (HNSCC) arising from the upper aerodigestive tract is a heterogeneous disease with varied pathological and therapeutic attributes that may modify the clinical presentation and outcomes [1]. Most patients with HNSCC present with locally advanced disease, many of whom need to be treated with concurrent chemoradiotherapy (CCRT). Patients with locally advanced head and neck squamous cell carcinoma (LAHNSCC) could receive CCRT as either surgery followed by adjuvant therapy for patients with oral cavity cancer (OCC) or as primary therapy with curative intent for non-oral cavity cancer (NOCC, oropharynx, hypopharynx, nasopharynx, and larynx). Owing to the tumor characteristics (location, size, and regional lymph node invasion), lifestyle habits, metabolic derangement, inflammatory factors induced by tumor and microenvironment, as well as significant in-field and systemic toxicity from CCRT, these patients often experience nutritional alterations and body weight loss before and during treatment [1,2,3,4,5].

Nutritional alterations and body weight loss in patients with LAHNSCC receiving CCRT is more specifically a change in the total body composition (TBC), commonly seen among cancer patients in response to varied situations such as aging, illness, metabolic strain, physiological change, and treatment [6,7]. Hence, monitoring the TBC change may offer an accurate assessment of nutritional/inflammatory alterations during CCRT [8,9,10]. Dual-energy X-ray absorptiometry (DXA), one of the gold standards for the evaluation of lean mass, fat mass, and bone mineral density of the total body, exactly measures each parameter with low radiation and costs [8,11,12].

Growing evidence has reported that DXA effectively assesses the TBC of LAHNSCC patients undergoing CCRT [3,13,14,15,16]. At the time of diagnosis, patients expressed lower values of lean body mass (LBM) and total fat mass (TFM) than healthy adults [15]. Throughout the CCRT course, DXA revealed incessant decreases in body weight (BW), LBM, TFM, and bone mineral content (BMC) [7,13,14,15]. Nevertheless, these results should be cautiously interpreted based on the following concerns: first, most studies have analyzed OCC and NOCC together. Since OCC and NOCC have different clinicopathologic characteristics and treatment modality sequences, the TBC change over the CCRT course may not be identical between the two subgroups. Second, because the OCC and NOCC subgroups have different intent CCRTs with varied irradiation fields, these reports did not assess the differential effects between adjuvant CCRT and primary CCRT on TBC change over the treatment course. Finally, certain pre-treated nutritional/inflammatory markers (NIMs) and treatment-related toxicities that are correlated with the malnourished status of patients with LAHNSCC undergoing CCRT [17,18] were not included in the analysis in the previous studies.

To address these concerns regarding the TBC changes of both LAHNSCC subgroups with different intent CCRTs, we carried out a prospective observational cohort study and enrolled patients with stage III, IVA, or IVB LAHNSCC who received standard CCRT. The patients received a supportive care program comprising biweekly dietitian appointments, sufficient daily calorie supplements, and adequate symptom control at a single institution. We stratified the participants into the OCC with adjuvant CCRT and NOCC with primary CCRT groups and analyzed the changes in TBC and different body regions between the two subgroups. Relevant information, which included clinicopathological variables, blood NIMs, and treatment-related toxicity profiles, was analyzed to identify potential factors contributing to the TBC change in both OCC and NOCC patients over the CCRT course.

## 2. Materials and Methods

### 2.1. Enrollment

We carried out this prospective cohort study between February 2015 and July 2019. The eligible patients had histologically proven LAHNSCC of the oral cavity, oropharynx, nasopharynx, hypopharynx, and larynx, which was classified into stages III (T1-2, N1 or T3, N0-1), IVA (T4a, N0-1 or T1-4a, N2), and IVB (any T, N3 or T4b, any N) according to the 7th edition of the American Joint Committee on Cancer (AJCC) staging system. Other eligibility criteria included age ≤75 years, negative for human papillomavirus test, Eastern Cooperative Oncology Group (ECOG) performance status score of less than 3, adequate hematopoietic or organ function, and could undergo CCRT. Patients were excluded if they had a positive expression of p16 in tumor specimens, or one of the following comorbid conditions: heart failure with New York Heart Association Classification IV, decompensated liver cirrhosis, end-stage renal disease, major gastrointestinal disorders, uncontrolled diabetes mellitus, autoimmune disorders, and active infections. Patients were also excluded if they were receiving regular medications that could significantly affect metabolism or body composition change, such as steroids or megestrol acetate. This study was approved by the Institutional Review Board of the Chang Gung Memorial Hospital, Taiwan (approval numbers: 103-3365A3 and 201700158B0) and was performed according to the Good Clinical Practice guidelines and the Declaration of Helsinki. All patients completed the written informed consent to participate.

### 2.2. Treatment Schedule

Patients with OCC received postoperative adjuvant CCRT after surgery if they had (1) one of the two major risk factors for extranodal extension or a positive surgical margin or (2) at least three of the following minor risk factors: pT4, pN1, close margin ≤4 mm, depth ≥10 mm, poor histologic grade differentiation, and vascular, lymph node, or perineural invasion. Patients with unresectable NOCC for organ preservation received primary CCRT alone. During CCRT, each patient received radiotherapy (RT) at a dose of 64–72 Gy in 32–36 fractions over 6–8 weeks, concurrently with chemotherapy administration using weekly cisplatin (40 mg/m^2^).

All patients had antiemetic medications for symptom control. They were referred to a nutrition support program comprising biweekly dietitian appointments, feeding tube placement if the BW loss was more than 5% during the CCRT course, suitable caloric supplementation, and blood transfusion as needed [19].

Following the European Society of Parenteral and Enteral Nutrition guideline [19,20], we offered 25–30 Kcal/kg/day wit% energy from carbohydrates:lipid = 60:40, and protein 1.0–1.5 g/kg/day for each patient during the CCRT course. We also provided oral nutritional supplements to patients who could not reach the required daily calorie intake via food. Hence, each patient could maintain the requirements of calculated energy and protein during the treatment course.

### 2.3. Clinicopathological Data and Blood NIMs

We collected clinicopathological data, including age, sex, body height and weight, ECOG performance status, comorbid illness, tumor sites, AJCC 7th edition of tumor node metastasis (TNM) stage, history of consumptions to smoking, alcohol and betel nut, treatment regimen, and toxicity profiles. The head and neck Charlson Comorbidity Index (HN-CCI) to assess the presence of comorbidities such as heart failure, pulmonary disease, cerebrovascular disease, peptic ulcers, liver disease, and diabetes was applied to score the severity of comorbid diseases [21]. Participants who currently smoked cigarettes or used to smoke in the past were considered smokers. Participants who reported consuming alcohol greater than 3 times per week were considered alcohol drinkers. Participants who reported consuming betel nut during the previous year were considered betel nut users. Body mass index (BMI) was defined as the weight in kilograms divided by height in square meters (kg/m^2^). The scores of patients generated subjective global assessment (PG-SGA) ranged from 0 to 35, with scores of 0–3 indicating well-nourished, 4–8 indicating moderately malnourished, and ≥9 indicating severely malnourished [22]. During the CCRT course, we defined the RT dose as the total radiation dose received by patients, the RT duration as the number of days the patients took to complete RT and the cisplatin dose as the cumulative dose of cisplatin administered.

We collected blood NIMs, including hemoglobin (Hb, g/dL), white blood cell count (WBC, 10^3^/mm^3^), platelet count (10^3^/mm^3^), total lymphocyte count (TLC) (10^3^/mm^3^), albumin (g/dL), and C-reactive protein (CRP) (mg/dL) before CCRT. TLC was calculated as WBC count (/mm^3^) × the percentage of lymphocytes in the blood.

### 2.4. Body Composition Assessment

Dual-energy fan-beam X-ray absorptiometry (Lunar iDXA, GE Medical System, Madison, WI, USA) was used to assess the TBC. The scanner software, according to body size and BMI, automatically selected the appropriate scan mode (standard, thin, or thick). Scans were analyzed using enCORE Software, version 15 (GE Lunar). Each participant was properly positioned set by the International Society for Clinical Densitometry guidelines [23]. The following parameters were acquired: LBM, TFM, appendicular skeletal mass (ASM, arm, and leg), and BMC. All three parameters were analyzed. ∆ indicates the interval changes in the above parameters before and after the treatment course.

All blood NIMs were completed within 1 week before CCRT. DXA-derived parameters were obtained 1 week before CCRT initiation and within 1 week after CCRT completion.

### 2.5. Statistical Analysis

We used SPSS (version 22.0; SPSS Inc., Chicago, IL, USA) to perform all statistical analyses. Based on a power of 80%, α error of 0.05, and the incidence rate of head and neck cancer in Taiwan, we calculated the minimum sample size to be 125. Patients with LAHNSCC might not complete the treatment course or data collection due to treatment intolerance, low compliance to medical advice, and inadequate family assistance. We then assumed that the attrition rate was 30%; therefore, the total number of patients that needed to be recruited was 169. All variables, both continuous and categorical, were examined and assessed for normality before analysis. Independent t-tests or Mann–Whitney tests for continuous variables and chi-square tests for categorical variables were used when appropriate. Paired t-test was used to detect the difference in BW and BMI, and Wilcoxon signed-rank test was used for body composition parameters between before and after CCRT. Analysis of variance using Bonferroni adjustments was used to detect the differences in treatment-interval changes in various body regions of body composition parameters (LBM, TFM, and BMC). Simple linear regression was used to relate age or BMI to the ∆LBM variable.

The associations between different clinicopathological variables, treatment-related factors, blood NIMs, and changes in DXA-derived body composition parameters (∆LBM, ∆TFM, and ∆BMC) were first analyzed by correlation analysis, independent *t*-tests, and analysis of variance (ANOVA) for continuous variables, and chi-square tests for categorical variables. All independent variables significantly associated with ∆LBM, ∆TFM, or ∆BMC (*p* ≤ 0.05) in the univariate analysis were included in the multivariable linear regression model analysis with forward stepwise selection. Variance inflation factors were used as variables to test for collinearity.

## 3. Results

### 3.1. Patient Characteristics

**Figure 1:**
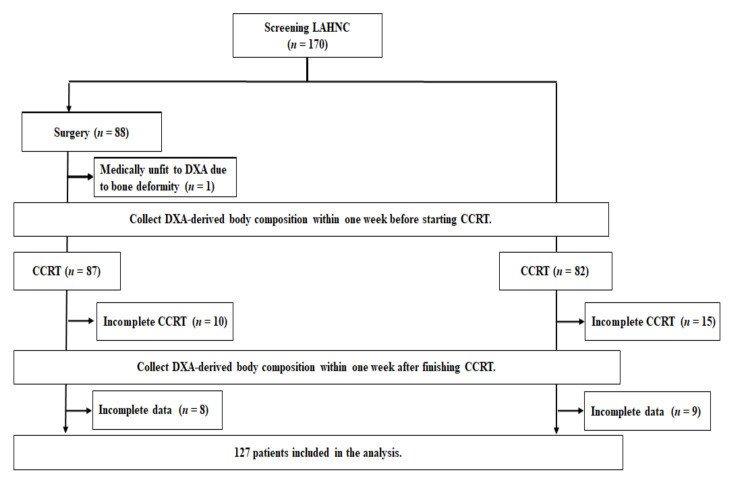
The CONSORT diagram. Incomplete CCRT was defined as patients who dropped out during the CCRT course or could not complete at least four cycles of weekly cisplatin (40 mg/m^2^) commitants with planned radiotherapy (64–72 Gy). Patients with incomplete data indicated that they failed to complete the required DXA examinations or scheduled blood tests; CCRT, concurrent chemoradiotherapy; DXA, dual-energy X-ray absorptiometry; LAHNC, locally advanced head and neck cancer.

We recruited a total of 170 patients with LAHNSCC. At the end of the study, 127 patients were eligible for analysis. The enrollment, allocation, treatment modality, and data collection details are displayed in the CONSORT diagram (Figure 1). The baseline and treatment characteristics of patients are shown in Table 1.

Male patients (96.9%) were predominant, and the mean age was 53.9 years. For OCC, the most common tumor subsite was tongue, followed by buccal mucosa and gingiva; for NOCC, it was hypopharynx, followed by tonsil and larynx. A high percentage of patients had exposure experience to smoking (90.6%), alcohol (74.8%), and betel nut (64.6%). The majority of tumors were non-metastatic TNM stage IV (92.1%), advanced tumor size (T3 + T4: 76.3%), and regional lymph invasion (N2 + N3: 66.1%). Among 127 patients, 77 (60.7%) had at least one comorbid illness, 56 (44.1%) underwent tracheostomy, and 108 (85%) had PG-SGA-defined malnourished status. The most common grade 3 or higher non-hematologic adverse effects were mucositis (25.2%) and infection (21.2%), while the hematologic counterpart was neutropenia (35.2%).

Certain baseline characteristics of patients with OCC receiving adjuvant CCRT were different from those of patients with NOCC receiving primary CCRT (Table 1). The OCC group had a higher platelet count and a higher proportion of patients with advanced tumor size status (≥T3 status), betel nut exposure, ECOG performance status of 2, and tracheostomy. In contrast, the NOCC subgroup had higher BMC, received more intense radiation treatment course (higher dose, more fractions, longer duration), and presented a higher percentage of patients with advanced regional lymph node invasion (≥N2 status) and grade ¾ infection toxicity. Even though each patient had the average daily calorie intake at 27.2 kcal/kg/day throughout the CCRT course, the OCC subgroup received more daily calorie intake over the treatment course than the NOCC subgroup (28.6 ± 8.6 vs. 25.7 ± 7.2 kcal/kg/day, *p* = 0.035).

### 3.2. Decreases in Body Weight, BMI, and DXA-Derived Parameters following CCRT Completion

Before treatment, the entire group had a mean BMI of 22.7 ± 4.9 kg/m^2^ and mean BW of 63.1 ± 12.1 kg. At the end of CCRT, the mean BMI was 21.6 ± 3.6 kg/m^2^ (mean 4.6% decline from the pretreatment, *p* < 0.001) and mean BW was 59.8 ± 10.6 kg (mean 4.7% decline from the pretreatment, *p* < 0.001). All body composition parameters were significantly decreased after completion of CCRT (LBM, 43.7 ± 5.8 vs. 41.0 ± 5.3 kg/m^2^, *p* < 0.001; TFM, 16.6 ± 7.7 vs. 15.6 ± 7.1 kg/m^2^, *p* < 0.001; BMC, 2.6 ± 0.4 vs. 2.5 ± 0.4 kg/m^2^, *p* < 0.001). On average, patients lost 5.8% of their LBM, 4.2% of TFM, and 2.8% of BMC over the course of CCRT. Despite the loss in all parameters at the end of treatment, the TBC ratio (the relative ratio between LBM, TFM, and BMC) remained unaffected over the CCRT course. The mean TBC was 68.2% LBM, 25.8% TFM, and 6.0% BMC at the start of CCRT. At the end of treatment, the TBC was almost identical, with 67.9% LBM, 25.9% TFM, and 6.2% BMC.

**Figure 2:**
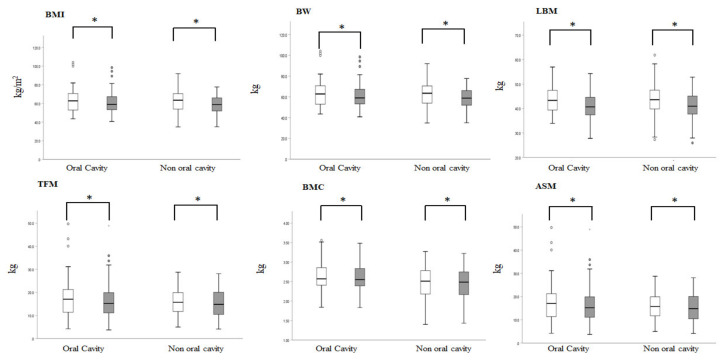
Box plots show the values of BMI, BW, and body composition parameters (LBM, TFM, BMC, ASM) at CCRT start (open box) and CCRT end (close box) in oral cavity cancer and non-oral cavity cancer. * denotes *p* < 0.05, considered significance between start and end. BMI, body mass index; BW, body weight; LBM, lean body mass; TFM, total fat mass; BMC, bone mineral content; ASM, appendicular skeletal mass; CCRT, concurrent chemoradiotherapy.

**Figure 3:**
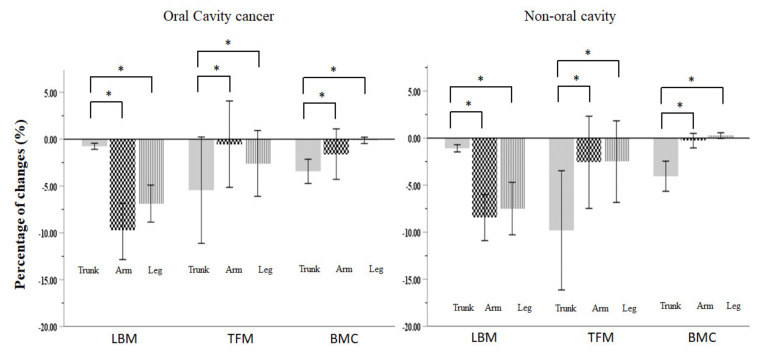
Percentage of treatment-interval changes in different body compartments of LBM, TFM, and BMC for both oral cavity cancer and non-oral cavity cancer. The percentage of change at each body compartment is determined by (posttreatment value−pretreatment value)/(pretreatment value) × 100%. * denotes *p* < 0.05, considered significant. LBM, lean body mass; TFM, total fat mass; BMC, bone mineral content; CCRT, concurrent chemoradiotherapy.

To further investigate the difference in TBC change throughout the CCRT course between the OCC and NOCC subgroups, we found that in accordance with the entire group, both subgroups showed similar and significant proportions of loss in BMI, BW, LBM, TFM, ASM, and BMC; the TBC ratios were unchanged: for the OCC subgroup, the mean TBC was 69.1% LBM, 26.8% TFM, and 4.1% BMC at the start of CCRT, with 68.7% LBM, 27.1% TFM, and 4.2% BMC at the end of CCRT; for the NOCC subgroup, the mean TBC was 70.1% LBM, 25.9% TFM, and 4.0% BMC at the start of CCRT, with 70.3% LBM, 25.6% TFM, and 4.21% BMC at the end of CCRT. LBM and TFM were lost in almost all the body regions. BMC was preferentially lost in the trunk area (Table 2 and Figure 2). Additionally, there was more muscle mass loss in the peripheral extremities (leg or arm) than in the central region (trunk) for LBM; in contrast, there was more fat mass loss in the central region than in the peripheral extremities for TFM. Interestingly, there was more TFM loss in the NOCC than in the OCC group (−6.1% vs. −2.6%, *p* = 0.042), particularly in the trunk (−9.8% vs. −5.4%, *p* = 0.046) and waist (−11.3% vs. −6.7%, *p* = 0.039). (Table 2 and Figure 3).

### 3.3. Factors Associated with Treatment-Interval Changes in LBM, TFM, and BMC following CCRT Completion

We simultaneously investigated the interactive effect among clinicopathologic variables, NIMs, and treatment-related factors on the interval changes of the body composition parameters over the CCRT course.

**Figure 4:**
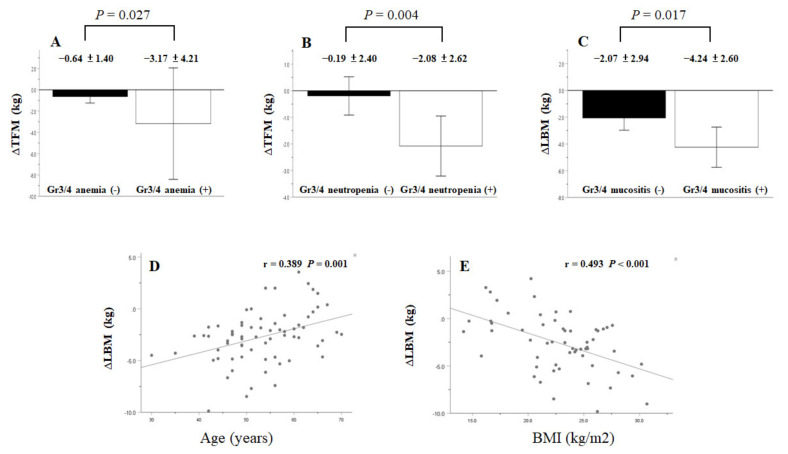
Oral cavity cancer patients with grade ¾ toxicity of anemia or neutropenia developed more TFM loss (**A**,**B**). Non-oral cavity cancer patients with grade ¾ mucositis toxicity developed more LBM loss (**C**). The association of age with ∆LBM in oral cavity cancer patients or BMI with ∆LBM in non-oral cavity cancer patients (**D**,**E**). Δ indicates a value obtained by subtracting the pre-CCRT value from the post-CCRT value. LBM, lean body mass; TFM, total fat mass; CCRT, concurrent chemoradiotherapy; BMI, body mass index.

For the OCC subgroup, the following variables were significant in the univariate analysis: age, mean daily calorie intake, and the pretreatment values of BMI, BW, and Hb for the interval LBM change (∆LBM); mean daily calorie intake, pretreatment values of BMI and BW, and grade ¾ toxicities of anemia, neutropenia, and thrombocytopenia for the interval TFM change (∆TFM); pretreatment values of BW and TLC for the interval BMC change (∆BMC). On multivariate analysis, the following variables were independent factors for the interval change of each body composition parameter: age and mean daily calorie intake for ∆LBM; mean daily calorie intake and grade ¾ toxicities of anemia and neutropenia for ∆TFM (Figure 4); pretreatment TLC alone for ∆BMC (Table 3).

For the NOCC subgroup, the following variables were significant in the univariate analysis: mean daily calorie intake, pretreatment values of BMI, BW, and albumin, and grade ¾ mucositis toxicity for ∆LBM; T status, pretreatment values of BMI and BW, mean daily calorie intake, and grade ¾ anemia toxicity for ∆TFM; pretreatment TLC and grade ¾ infection toxicity for ∆BMC. On multivariate analysis, the following variables were independent factors for the interval change of each body composition parameter: mean daily calorie intake, BMI, and grade ¾ mucositis toxicity for ∆LBM (Figure 4); mean daily calorie intake for ∆TFM; pretreatment TLC alone for ∆BMC (Table 4).

## 4. Discussion

The current study stratified patients with LAHNSCC into two subgroups according to tumor location and CCRT setting based on different patient characteristics and therapeutic intent: OCC with postoperative adjuvant CCRT and NOCC with primary curative intent CCRT. Postoperative adjuvant CCRT is aimed at theoretically tumor-free OCC patients, whose TBC changes result from treatment alone; in contrast, primary curative-intent CCRT is administered to NOCC patients, whose TBC change is the summative effect of both cancer and treatment. Under adequate supportive care, including dietary calorie monitoring and nutrition counseling, we found that, in line with previous reports [7,13,14,24], BW, BMI, and body composition parameters (LBM, TFM, and BMC) were progressively lost over the treatment course for both subgroups as well as the entire group. Furthermore, almost all body regions of LBM and TFM presented loss in different proportions, particularly for more muscle mass loss in the peripheral extremities than in the trunk for LBM, but more fat mass loss in the trunk than in the peripheral extremities for TFM. There was more TFM loss in the NOCC than in the OCC subgroup, and BMC loss preferentially occurred in the trunk region. After adjusting for covariates, including clinicopathological variables, pretreatment blood NIMs, and treatment-related factors, we showed that different independent factors correlated with the interval changes of each body composition parameter over the CCRT course in both OCC and NOCC subgroups. To our knowledge, this prospective study is the first to use DXA to assess the difference in TBC change between patients with OCC who received postoperative adjuvant CCRT and those with NOCC who received primary CCRT.

The TBC changes included alterations in the LBM, TFM, and BMC. More than 70% of the body weight loss following CCRT is associated with LBM loss [7]. Such a lean muscle loss has clinical relevance and has a negative impact on survival and locoregional control in patients with HNSCC [14,15,25]. The LBM loss in patients with LAHNSCC undergoing CCRT is mediated by elevated levels of reactive oxygen species and pro-inflammatory cytokines, activation of proteolysis machinery including ubiquitin-dependent, calcium-dependent, and autophage/lysosome systems, decreased mitochondrial biogenesis and mass, and alteration of lipid metabolism [26,27]. The above mechanisms are integrated into two essential clinical factors participating in LBM loss during CCRT: patient characteristics such as age [28], tumor features [29], comorbidity [30], performance status [31], lifestyle habits [32,33,34], pretreatment nutritional status [35]; treatment-related attributes including RT treatment (dose, fraction, and duration), chemotherapy regimen [26,36,37], daily calorie delivered over the treatment course [9,37], and CCRT-associated toxicities [38,39]. The multivariate analysis in the present study showed different factors contributing to LBM loss for the OCC and NOCC subgroups: age and mean daily calorie intake for the OCC; mean daily calorie intake, BMI, and grade ¾ mucositis toxicity for the NOCC. Our data suggest that treatment-interval LBM loss should be considered as a multifactorial muscle wasting status for patients with LAHNSCC receiving CCRT.

TFM loss is frequently accompanied by LBM loss during CCRT in patients with LAHNSCC [7,13,14,15,40]. Willemsen et al. showed that early fat loss (TFM loss over 1% in the first 3 weeks of therapy) had no significant effect on the overall survival of patients with HNSCC undergoing CCRT [40]. Prowrozek et al. reported that the 13041A/G polymorphism of the *PLNI1* gene encoding perilipin, a regulatory protein for the balance between fat storage and decomposition, has a higher predictive value for the development of severe TFM loss in LAHNSCC treated by CCRT [41]. Furthermore, the serum level of miRNA21, a key microRNA gene involved in fat metabolism, could be modulated by CCRT in patients with LAHNSCC [42,43]. A growing body of research has explored the clinical relevance of TFM loss during CCRT, but its implication in prognostic outcomes and interplay with other body composition parameters in patients with LAHNSCC remains to be examined. Similar to LBM loss, our data support the notion that TFM loss during CCRT could be affected by multiple factors, such as daily calorie intake and treatment-related toxicities.

TFM loss from the adipose tissue of patients with LAHNSCC over the CCRT course could be considered as an additive or synergetic result of decreased lipogenesis, increased lipolysis, and insufficient calorie intake [37]. Garcia et al. found that cisplatin-treated healthy male mice fed ad lib showed increased lipolysis in adipose tissue, elevated β-oxidation in liver and adipose tissue, and suppressed lipogenesis in the liver, adipose tissue, and muscle; however, mice with reduced food intake in pair-fed experiments expressed lower activities of enzymes involved in lipolysis and lipogenesis in adipose tissue, liver, and muscle. Garcia’s findings suggested that modification of food intake may interfere with cisplatin-mediated fat metabolism [37]. Jager-Wittenaar et al. also reported that patients with HNSCC could maintain total body fat mass by providing nutritional support with sufficient energy (35 kcal/kg/day) and protein intake (1.5 g/kg/day) throughout the treatment course [14]. TFM loss may precede LBM loss in patients with cancer during the cachectic process [44]. Hence, sufficient calorie intake during the treatment may reduce adipose tissue loss, likely resulting from reduced treatment-induced anorexia effect and lipid catabolism, the result of which may help patients improve loss of body muscle mass. Our data demonstrated that mean daily calorie intake significantly correlated with both LBM and TFM loss, and less energy intake accompanied with more TFM loss was noted in the NOCC group than in the OCC group. These findings lend further support to the intimate correlation between daily calorie intake and the maintenance of body muscle and fat mass during CCRT.

In addition to daily calorie intake during CCRT, certain treatment-associated grade ¾ toxicities may intensify LBM or TFM loss [45]. Our data showed that patients in the OCC subgroup who developed grade ¾ toxicity with either anemia or neutropenia lost more fat mass, and those in the NOCC with grade ¾ mucositis lost more muscle mass (Table 3 and Table 4; Figure 4). These toxicities may reduce food intake, enhance inflammatory response, or increase lipid and protein catabolism, which consequently worsens the nutritional status and induces muscle or fat mass loss [38,39,46]. Thus, it is reasonable that appropriate control of treatment toxicity could prevent LBM and TFM loss during anti-cancer therapy [38].

Although LBM loss accounts for over 70% BW loss in patients with LAHNSCC during CCRT, most studies have explored the predictive effects of both age and BMI on BW loss rather than on LBM loss; however, the results regarding BW loss prediction have been conflicting [17,45,47,48,49]. From our own perspective, these inconsistent observations could be ascribed to retrospective design, enrollment heterogeneity (variations in tumor stage, mixed head and neck cancer entities, and treatment modalities), the preference for tube feeding from patients and healthcare professionals, the classification of aging and BMI by different ranges, and lack of comprehensive risk factor analysis. We considered the confounding effects of the above-mentioned variables and a better survival outcome prediction of treatment-interval LBM loss as compared to BW loss [1], and then designed the current prospective study recruiting patients with homogenous backgrounds that were stratified into the OCC and NOCC subgroups. Our data demonstrated that after adjustment for all possible covariates, age presented a positive correlation for the OCC subgroup, and BMI had a negative correlation with treatment-interval LBM loss (Table 3 and Table 4; Figure 4). We further investigated the correlation between age or BMI and all clinicopathological variables, treatment factors, and NIMs before treatment, and found that age was negatively correlated with resting metabolic rate calculated using the Harris–Benedict equation [50] in the OCC subgroup, and BMI was positively correlated with resting metabolic rate (Table S1). de Carvalho et al. also reported that a higher BMI in patients with LAHNSCC receiving CCRT experienced higher resting energy expenditure, as determined by indirect calorimetry [51]. We also observed that in the NOCC group, BMI was positively correlated with albumin level and negatively correlated with daily calorie intake (Table S1). Hence, patients with high BMI had better nutritional status and might have less aggressiveness in the daily calorie intake demand than those with low BMI; consequently, less daily calorie intake may lead to greater muscle mass loss during CCRT. Taken together, we speculate that resting metabolic rate and energy intake might be the key factors in determining the effects of age and BMI on LBM loss during CCRT.

We also showed significant BMC loss during CCRT, particularly in the trunk area, and pretreatment TLC in the peripheral blood was the only contributing factor and presented a negative association with this loss. TLC successfully predicts outcomes among patients with aging, chronic illness, and cancers and is closely associated with bone health [52,53,54,55]. It is likely that the process of BMC loss during CCRT resembles the development of osteoporosis, which is closely related to inflammatory diseases such as autoimmune disorders, inflammatory bowel disease, and chronic obstructive pulmonary disease. On the one hand, inflammation is mainly mediated by inflammatory cytokines, including tumor necrosis factor-α, interleukin (IL)-1, IL-6, and IL-17 produced from peripheral blood lymphocytes and subsequently induces the generation of the primary disease. On the other hand, it could disrupt the balance of skeletal metabolism through the RANK/RANKL/OPG signaling pathway, leading to osteoclast activation and bone resorption [55]. As such, pretreatment TLC may represent an indicator of inflammation severity before CCRT, which may deteriorate bone loss in patients with LAHNSCC during the treatment course.

In this study, patients developed more muscle mass loss in the peripheral extremities than in the trunk for LBM, but more fat mass loss in the trunk than in the peripheral extremities for TFM. Our results were comparable to those reported by Fouladium et al., who applied DXA to assess the TBC change in 311 patients with metastatic cancers, predominantly gastrointestinal and hepatobiliary tract tumors, who received no active anti-cancer treatment during the study period [9]. Based on Fouladium’s results and our results, certain hypothetical mechanisms whereby cancer or treatment mediates such a preferential loss of LBM and TFM in different body compartments are proposed. First, under stress, catecholamine released from the activated sympathetic nervous system modulates lipolysis. Catecholamine could subsequently trigger downstream β-adrenergic receptors to perform lipolysis or bind to α2- adrenergic receptors to counteract lipolysis [56]. The α2- adrenergic receptor is expressed more in subcutaneous adipose tissue than in visceral adipose tissue [56]. When subjected to BW reduction, obese individuals have more loss in visceral adipose tissue than subcutaneous adipose tissue [56]. Preferential TFM loss in the central area could be the result of lipolysis regulated by catecholamine binding to α2- adrenergic receptor and β-adrenergic receptors among various fat tissue compartments. Second, inflammatory cytokines such as IL-1β, tumor necrosis factor-α, and IL-6, all of which actively participate in the process of lipogenesis and lipolysis, are expressed more in visceral adipose tissue than in subcutaneous adipose tissue in obese people [57]. High serum IL-6 levels are associated with arm and leg muscle loss in aging people [58]. Insulin resistance and serum leptin levels were also associated with appendicular skeletal muscle loss in elderly and hemodialysis patients [59,60]. The serum levels of cytokines, insulin, ghrelin, leptin, adiponectin, cortisol, and thyroid hormones were altered during cancer progression and the CCRT course [9,51]. Accordingly, it is possible that protein and lipid metabolism in different body regions may be diverse and influenced by these cytokines and metabolic hormones. Lastly, regional metabolic differences were noted among varied muscular compartments, particularly in the protein synthesis of the appendicular skeletal muscle area [61]. Altogether, the possible mechanisms pertaining to the preferential loss of LBM and TFM in different body compartments of patients with LAHNSCC undergoing CCRT are complex and multifactorial.

The major limitation of the current study is that patients were male dominant and recruited from the Taiwanese population. Our results should be cautiously extrapolated to the female gender, non-Taiwanese patients, different treatment schedules, and nutrition support programs. Fluctuating fluid status during treatment may raise the question as to whether LBM loss detected by DXA may just be a reflection of volume status. Going et al. analyzed body composition changes in 17 cancer patients by DXA during a dehydration-rehydration protocol and found that BW change due to small fluid loss and gain was correlated with LBM, but not with TFM and BMC, which were not affected by the changes in hydration [62]. We also arranged serial computed tomography (CT) scans of the abdomen to assess the fluid status and body composition during CCRT. The results showed no abnormal fluid accumulation over the treatment course and a significant correlation in the LBM index by DXA and skeletal muscle index by CT (Figure S1). We were confident in the overall accuracy of the body composition parameters investigated by DXA in the current study.

## 5. Conclusions

The current prospective observational study demonstrates the difference in treatment-interval change in TBC between OCC patients with postoperative adjuvant CCRT and NOCC with primary CCRT. Different factors are independently associated with the interval changes in each body composition parameter over the course of CCRT. Enrollment with homogeneous tumor entities and CCRT settings is essential in nutrition-oriented clinical trials of patients with head and neck cancer.

## Figures and Tables

**Table 1:** Baseline and treatment characteristics of 127 LAHNSCC patients treated by CCRT.

Variables	Total	Oral Cavity with Adjuvant CCRT	Non-Oral Cavity with Primary CCRT	
Variables Expressed as Numbers (%) or Mean ± SD				*p* Value *
**Included patient number**	127 (100)	69 (54.3)	58 (45.7)	
**Age (years)**	53.9 ± 8.8	53.2 ± 8.4	54.6 ± 9.2	0.374
**Sex (male:female)**	123 (96.9):4 (3.1)	68 (98.6):1 (1.4)	55 (94.8):3 (5.2)	0.231
**Tumor subsites**				
Buccal mucosa	20 (15.7)	20 (29.0)		
Tongue	28 (22.0)	28 (40.6)		
Gingiva	13 (10.2)	13 (18.9)		
Mouth floor	3 (2.4)	3 (4.3)		
Retromolar	2 (1.6)	2 (2.9)		
Lip	2 (1.6)	2 (2.9)		
Hard palate	1 (0.8)	1 (1.4)		
Tonsil	13 (10.2)		13 (22.4)	
Tongue base	6 (4.7)		6 (10.3)	
Soft palate	3 (2.4)		3 (5.2)	
Hypopharynx	24 (18.9)		24 (41.4)	
Larynx	8 (6.3)		8 (13.8)	
Nasopharynx	4 (3.2)		4 (6.9)	
**TNM Stage (III:IVA:IVB)**	10 (7.9):87 (68.5):30 (23.6)	4 (5.8):50 (72.5):15 (21.7)	6 (10.3):37 (63.8):15 (25.9)	0.497
**Tumor size**				0.004 *
T0	2 (1.6)	0 (0.0)	2 (3.4)	
T1	7 (5.5)	2 (2.9)	5 (8.6)	
T2	21 (16.5)	6 (8.7)	15 (25.9)	
T3	21 (16.5)	11 (15.9)	10 (17.2)	
T4a	64 (50.4)	45 (65.2)	19 (32.8)	
T4b	12 (9.4)	5 (7.3)	7 (12.1)	
**LN involvement**				0.035 *
N0	25 (19.7)	21 (30.4)	4 (6.9)	
N1	18 (14.2)	9 (13.1)	9 (15.5)	
N2	64 (50.4)	29 (42.0)	35 (60.4)	
N3	20 (15.7)	10 (14.5)	10 (17.2)	
**Histological grade (1:2:3)**	11 (8.7):86 (67.7):30 (23.6)	8 (11.6):51 (73.9):10 (14.5)	3 (5.2):35 (60.3):20 (34.5)	0.021 *
**Smoking (no:yes)**	12 (9.4):115 (90.6)	6 (8.7):63 (91.3)	6 (10.3):52 (89.7)	0.752
**Alcohol (no:yes)**	32 (25.2):95 (74.8)	18 (26.1):51 (73.9)	14 (24.1):44 (75.9)	0.801
**Betel nut (no:yes)**	45 (35.4):82 (64.6)	16 (23.2):53 (76.8)	29 (50.0):29 (50.0)	0.002 *
**HN-CCI (0:1:2: ≥3)**				0.408
0	50 (39.4)	29 (42.1)	21 (36.2)	
1	31 (24.4)	15 (21.7)	16 (27.6)	
2	14 (11.0)	6 (8.7)	8 (13.8)	
≥3	31 (25.2)	19 (27.5)	13 (22.4)	
**ECOG performance status (0:1:2)**	10 (7.9):110 (86.6):7 (5.5)	2 (2.9):61 (86.4):6 (8.6)	8 (13.8):49 (84.5):1 (1.7)	0.046 *
**Tracheostomy (no:yes)**	71 (55.9):56 (44.1)	23 (33.3):46 (66.7)	48 (82.8):10 (17.2)	<0.001 *
**PG-SGA (well:moderate:severe)**	19 (15.0):73 (57.4):35 (27.6)	13 (18.8):38 (55.1):18 (26.1)	6 (10.4):35 (60.3):17 (29.3)	0.408
**Anthropometric and biochemical data before CCRT**				
BW (kg)	63.0 ± 12.1	63.6 ± 12.6	62.4 ± 11.7	0.583
BMI (kg/m^2^)	22.7 ± 4.0	22.7 ± 4.3	22.8 ± 3.9	0.961
Hb (g/dL)	11.9 ± 1.6	11.7 ± 1.5	12.1 ± 1.8	0.157
WBC (×10^3^ cells/mm^3^)	7.2 ± 2.7	7.3 ± 2.5	7.1 ± 2.9	0.811
Platelet count (×10^3^/mm^3^)	30.1.8 ± 127.9	341.1 ± 148.4	254.9 ± 76.2	<0.001 *
TLC (×10^3^ cells/mm^3^)	1.7 ± 0.6	1.6 ± 0.6	1.8 ± 0.7	0.134
Albumin (g/dL)	3.8 ± 0.5	3.8 ± 0.6	3.8 ± 0.5	0.578
CRP (mg/dL)	14.2 ± 11.6	11.2 ± 1.8	11.9 ± 6.1	0.260
ALT (U/L)	23.0 ± 13.2	24.3 ± 1.6	21.4 ± 1.7	0.223
Creatinine (mg/dL)	0.95 ± 1.28	0.81 ± 0.03	1.12 ± 0.24	0.184
eGFR (mL/min/1.73 m^2^)	108.8 ± 35.8	113.9 ± 4.3	102.7 ± 4.6	0.079
**DXA-related measurements before CCRT**				
LBM (kg)	43.7 ± 5.9	43.8 ± 5.1	43.6 ± 6.7	0.868
TFM (kg)	16.6 ± 7.6	17.0 ± 8.8	16.1 ± 5.9	0.516
ASM (kg)	18.6 ± 5.4	18.4 ± 3.0	18.7 ± 3.7	0.582
BMC (kg)	2.55 ± 0.38	1.35 ± 0.44	1.36 ± 0.55	0.023 *
**Mean daily calorie intake during CCRT (kcal/kg/day)**	27.2 ± 8.1	28.6 ± 8.6	25.7 ± 7.2	0.035 *
**CCRT regimen**				
Radiotherapy				
Dose (Gy)	66.8 ± 4.4	64.3 ± 3.8	69.9 ± 3.0	<0.001 *
Fractions	32.6 ± 1.7	32.0 ± 1.5	33.4 ± 1.4	<0.001 *
Duration (days)	49.7 ± 6.6	48.0 ± 4.8	51.6 ± 7.8	0.003 *
Cisplatin dose (mg/m^2^)	227.8 ± 47.1	238.5 ± 45.5	215.0 ± 64.0	0.01 *
**Toxicity during CCRT**				
Non-hematologic (any Grade:Grade ¾)				
Dermatitis	121 (89.8):6 (4.7)	66 (89.8):3 (4.3)	55 (89.8):3 (5.2)	0.827
Pharyngitis	52 (40.9):14 (11.1)	24 (34.8):4 (5.7)	28 (48.3):10 (17.2)	0.082
Infection	31 (24.4):27 (21.2)	13 (18.8):10 (14.4)	18 (31.4):17 (29.3)	0.042 *
Mucositis	46 (36.2):32 (25.2)	27 (39.1):18 (26.0)	19 (32.8):14 (24.1)	0.653
Emesis	61 (48.0):10 (7.5)	33 (47.8):6 (8.7)	28 (48.3):4 (6.9)	0.708
Hematologic (any Grade:Grade ¾)				
Anemia	123 (96.9):12 (9.5)	66 (95.8):5 (7.2)	57 (98.3):7 (12.0)	0.355
Neutropenia	102 (80.3):45 (35.5)	57 (82.6):23 (33.3)	45 (77.6):22 (38.0)	0.589
Thrombocytopenia	85 (66.9):12 (9.5)	42 (60.9):4 (5.7)	43 (74.1):8 (13.8)	0.125

* Compare the value difference between the oral cavity and non-oral cavity for each variable. *p* < 0.05 represents statistical significance. Abbreviations: LAHNSCC, locally advanced head and neck squamous cell carcinoma; CCRT, concurrent chemoradiotherapy; SD, standard deviation; LN, lymph node; HN-CCI, Charlson Comorbidity Index; ECOG, Eastern Cooperative Oncology Group; PG-SGA, patient-generated subjective global assessment; BW, body weight; BMI, body mass index; Hb, hemoglobin; WBC, white blood cell; TLC, total lymphocyte count; CRP, C-reactive protein; ALT, alanine aminotransferase; eGFR, estimated glomerular filtration rate; DXA, dual-energy X-ray absorptiometry; LBM, lean body mass; TFM, total fat mass; ASM, appendicular skeletal mass; BMC, bone mineral content.

**Table 2:** Treatment-interval changes in different body regions of DXA-derived body composition parameters in 127 LAHNSCC patients stratified by tumor locations and CCRT settings.

	Oral Cavity with Adjuvant CCRT				Non-Oral Cavity with Primary CCRT			
Variables Expressed as Mean ± SD, kg	CCRT Starts	CCRT Ends	% Change	*p* Value *	CCRT Starts	CCRT Ends	% Change	*p* Value *
**BW**	63.6 ± 12.6	60.7 ± 11.2	−4.1	<0.001	62.4 ± 11.7	58.7 ± 9.9	−3.7	<0.001
**BMI**	22.7 ± 4.3	21.8 ± 3.9	−3.8	<0.001	22.8 ± 3.9	21.4 ± 3.3	−5.5	<0.001
**LBM**	43.8 ± 5.1	41.1 ± 5.0	−6.1	<0.001	43.6 ± 6.7	41.0 ± 5.8	−5.6	<0.01
Arm	5.2 ± 0.8	4.6 ± 0.8	−9.8	<0.001	5.2 ± 1.1	4.7 ± 0.9	−8.4	<0.001
Leg	13.2 ± 2.4	12.2 ± 2.2	−6.8	<0.001	13.4 ± 2.7	12.4 ± 2.4	−7.5	<0.001
Trunk	21.9 ± 2.2	21.1 ± 2.0	−0.7	<0.001	21.4 ± 3.0	20.2 ± 2.7	−1.1	<0.001
Waist	3.2 ± 0.4	3.1 ± 0.3	−3.8	<0.001	3.2 ± 0.5	2.9 ± 0.4	−5.7	<0.001
Hip	6.2 ± 0.9	5.8 ± 0.8	−6.0	<0.001	6.3 ± 1.2	5.7 ± 1.1	−8.0	<0.001
**TFM**	17.0 ± 8.8	16.2 ± 8.1	−2.6	0.012	16.1 ± 5.9	14.9 ± 5.6	−6.1	0.01
Arm	6.2 ± 0.9	5.8 ± 0.8	−2.5	<0.001	6.3 ± 1.2	5.7 ± 1.1	−2.6	<0.001
Leg	4.6 ± 2.4	4.4 ± 2.2	−2.5	0.018	4.2 ± 1.5	4.0 ± 1.4	−2.5	0.052
Trunk	9.7 ± 5.7	8.8 ± 5.2	−5.4	<0.001	9.3 ± 3.9	8.1 ± 3.6	−9.8	<0.001
Waist	1.6 ± 1.1	1.4 ± 0.9	−6.7	<0.001	1.5 ± 0.7	1.2 ± 0.6	−11.3	<0.001
Hip	2.3 ± 1.2	2.2 ± 1.2	−2.1	0.084	2.2 ± 0.8	2.1 ± 0.8	−4.4	0.019
**BMC**	2.6 ± 0.3	2.5 ± 0.3	−1.3	<0.001	2.5 ± 0.4	2.4 ± 0.4	−0.7	<0.001
Arm	0.39 ± 0.05	0.39 ± 0.07	−1.6	0.233	0.37 ± 0.07	0.37 ± 0.07	−0.3	0.291
Leg	0.94 ± 0.12	0.94 ± 0.13	−0.1	0.689	0.88 ± 0.14	0.88 ± 0.13	+0.2	0.081
Trunk	0.75 ± 0.14	0.72 ± 0.14	−3.4	<0.001	0.68 ± 0.17	0.65 ± 0.17	−4.1	<0.001
Waist	0.05 ± 0.01	0.05 ± 0.02	−0.9	0.339	0.04 ± 0.01	0.04 ± 0.02	+1.0	0.463
Hip	0.24 ± 0.04	0.24 ± 0.03	−0.2	0.416	0.22 ± 0.04	0.22 ± 0.03	−0.1	0.963

* Compare the value difference between CCRT starts and CCRT ends for each variable; *p* < 0.05 represents statistical significance. Abbreviations: DXA, dual-energy X-ray absorptiometry; LAHNSCC, locally advanced head and neck squamous cell carcinoma; CCRT, concurrent chemoradiotherapy; SD, standard deviation; BW, body weight; BMI, body mass index; LBM, lean body mass; TFM, total fat mass; BMC, bone mineral content.

**Table 3:** Univariate and multivariate associations of clinicopathologic variables, treatment-related factors, nutritional and inflammatory markers with changes of body composition parameters over the CCRT course in 69 patients with oral cavity cancer undergoing adjuvant CCRT.

Variables	ΔLBM			ΔΤFM			ΔBMC		
	Univariate	Multivariate		Univariate	Multivariate		Univariate	Multivariate	
	*p*-Value *	Coefficient (95% CI)	*p*-Value *	*p*-Value *	Coefficient (95% CI)	*p*-Value *	*p*-Value *	Coefficient (95% CI)	*p*-Value *
***Clinicopathologic factor***									
Age	0.001 *	0.090 (0.028~0.152)	0.005 *	0.237			0.596		
Sex	0.411			0.594			0.382		
TNM stage (III vs. IVA vs. IVB)	0.594			0.804			0.436		
T status (T1-2 vs. T3-4)	0.630			0.647			0.968		
N status (N0-1 vs. N2-3)	0.486			0.899			0.492		
Histologic grade (1 vs. 2 vs. 3)	0.906			0.850			0.552		
Smoking (no vs. yes)	0.703			0.666			0.455		
Alcohol (no vs. yes)	0.419			0.516			0.062		
Betel nut (no vs. yes)	0.803			0.374			0.483		
ECOG performance status (0:1:2)	0.480			0.433			0.310		
HN-CCI (0 vs. 1 vs. 2 vs. ≥3)	0.556			0.707			0.552		
Tracheostomy (no vs. yes)	0.395			0.509			0.609		
Mean daily calorie intake during CCRT	0.001 *	0.102 (0.043~0.162)	0.001 *	<0.001 *	0.133 (0.073~0.193)	<0.001 *	0.141		
***Treatment-associated factors***									
CCRT Regimen									
RT dose	0.958			0.658			0.192		
RT fractions	0.587			0.368			0.237		
RT duration (days)	0.474			0.670			0.855		
Cisplatin dose	0.717			0.731			0.815		
Grade ¾ toxicity									
Dermatitis (ref: yes)	0.255			0.889			0.097		
Pharyngitis (ref: yes)	0.713			0.786			0.055		
Infection (ref: yes)	0.283			0.958			0.313		
Mucositis (ref: yes)	0.611			0.378			0.601		
Emesis (ref: yes)	0.351			0.160			0.569		
Anemia (ref: yes)	0.988			0.037 *	2.550 (0.553~0.538)	0.014 *	0.456		
Neutropenia (ref: yes)	0.535			0.004 *	1.675 (0.450~2.899)	0.009 *	0.931		
Thrombocytopenia (ref: yes)	0.271			0.048 *			0.944		
***NIMs before CCRT***									
PG-SGA (well: moderate: severe)	0.728			0.473			0.706		
BMI	0.038 *			<0.001 *			0.061		
BW	0.023 *			<0.001 *			0.030 *		
Hb	0.027 *			0.469			0.848		
WBC	0.990			0.252			0.145		
Platelet	0.071			0.353			0.199		
TLC	0.188			0.701			0.005 *	−0.021 (−0.080~−0.01)	0.031 *
Albumin	0.350			0.330			0.983		
CRP	0.195			0.870			0.254		

* represents a significant *p*-value. Abbreviations: CCRT, concurrent chemoradiotherapy; HN-CCI, Charlson Comorbidity Index; ECOG, Eastern Cooperative Oncology Group; PG-SGA, patient generated subjective global assessment; RT, radiotherapy; NIMs, nutritional/inflammatory markers; BW, body weight; BMI, body mass index; Hb, hemoglobin; WBC, white blood cell; TLC, total lymphocyte count; CRP, C-reactive protein; LBM, lean body mass; TFM, total fat mass; BMC, bone mineral content. Δ indicates a value obtained by subtracting the pre-CCRT value from the post-CCRT value; CI, confidence interval.

**Table 4:** Univariate and multivariate associations of clinicopathologic variables, treatment-related factors, nutritional and inflammatory markers with changes of body composition parameters over the CCRT course in 58 patients with non-oral cavity cancer undergoing primary CCRT.

Variables	ΔLBM			ΔΤFM			ΔBMC		
	Univariate	Multivariate		Univariate	Multivariate		Univariate	Multivariate	
	*p*-Value *	Coefficient (95% CI)	*p*-Value *	*p*-Value *	Coefficient (95% CI)	*p*-Value *	*p*-Value *	Coefficient (95% CI)	*p*-Value *
***Clinicopathologic factors***									
Age	0.489			0.195			0.168		
Sex	0.154			0.772			0.627		
TNM stage (III vs. IVA vs. IVB)	0.565			0.443			0.538		
T status (T1-2 vs. T3-4)	0.389			0.017 *			0.563		
N status (N0-1 vs. N2-3)	0.523			0.836			0.693		
Histologic grade (1 vs. 2 vs. 3)	0.658			0.265			0.590		
Smoking (no vs. yes)	0.626			0.203			0.758		
Alcohol (no vs. yes)	0.234			0.071			0.919		
Betel nut (no vs. yes)	0.661			0.249			0.435		
ECOG performance status (0:1:2)	0.336			0.627			0.496		
HN-CCI (0 vs. 1 vs. 2 vs. ≥3)	0.802			0.574			0.098		
Tracheostomy (no vs. yes)	0.187			0.080			0.271		
Mean daily calorie intake during CCRT	0.005	0.167 (0.016~0.257)	0.001	<0.001 *	0.148 (0.044~0.250)	0.006 *	0.914		
***Treatment-associated factors***									
CCRT Regimen									
RT dose	0.874			0.792			0.862		
RT fractions	0.567			0.533			0.806		
RT duration (days)	0.474			0.670			0.855		
Cisplatin dose	0.534			0.173			0.658		
Grade ¾ toxicity									
Dermatitis (ref: yes)	0.262			0.111			0.985		
Pharyngitis (ref: yes)	0.706			0.950			0.247		
Infection (ref: yes)	0.959			0.752			0.038 *		
Mucositis (ref: yes)	0.017 *	2.538 (1.038~4.038)	0.001	0.796			0.628		
Emesis (ref: yes)	0.706			0.354			0.495		
Anemia (ref: yes)	0.759			0.040 *			0.158		
Neutropenia (ref: yes)	0.958			0.251			0.178		
Thrombocytopenia (ref: yes)	0.565			0.186			0.976		
***NIMs before CCRT***									
PG-SGA (well: moderate: severe)	0.133			0.473			0.706		
BMI	<0.001 *	−0.367 (−0.556~−0.177)	0.001	0.030 *			0.914		
BW	<0.001 *			0.017 *			0544		
Hb	0.740			0.304			0.958		
WBC	0.150			0.689			0.229		
Platelet	0.098			0.469			0.171		
TLC	0.290			0.897			0.040 *	−0.025 (−0.040~−0.009)	0.029 *
Albumin	0.022 *			0.464			0.380		
CRP	0.101			0.624			0.907		

* represents a significant *p*-value. Abbreviations: CCRT, concurrent chemoradiotherapy; HN-CCI, Charlson Comorbidity Index; ECOG, Eastern Cooperative Oncology Group; PG-SGA, patient-generated subjective global assessment; RT, radiotherapy; NIMs, nutritional/inflammatory markers; BW, body weight; BMI, body mass index; Hb, hemoglobin; WBC, white blood cell; TLC, total lymphocyte count; CRP, C-reactive protein; LBM, lean body mass; TFM, total fat mass; BMC, bone mineral content. **Δ** indicates a value obtained by subtracting the pre-CCRT value from the post-CCRT value.

## Data Availability

The data presented in this study are available on request from the corresponding author.

## Supplementary Materials

The following are available online at https://www.mdpi.com/article/10.3390/nu13092969/s1, Figure S1: Significant correlation between LBM index assessed by DXA and skeletal muscle index assessed by CT scan of abdomen, Table S1: The associations between age, BMI, clinicopathologic variables, treatment-related factors, and nutritional/inflammatory markers in 127 LAHNSCC patients before CCRT.

## References

[1] Baxi S.S., Schwitzer E., Jones L.W. (2016). A review of weight loss and sarcopenia in patients with head and neck cancer treated with chemoradiation. Cancers Head Neck.

[2] Alshadwi A., Nadershah M., Carlson E.R., Young L.S., Burke P.A., Daley B.J. (2013). Nutritional Considerations for Head and Neck Cancer Patients: A Review of the Literature. J. Oral Maxillofac. Surg..

[3] Capozzi L.C., McNeely M., Lau H.Y., Reimer R.A., Giese-Davis J., Fung T.S., Culos-Reed S.N. (2016). Patient-reported outcomes, body composition, and nutrition status in patients with head and neck cancer: Results from an exploratory randomized controlled exercise trial. Cancer.

[4] Couch M., Lai V., Cannon T., Guttridge D., Zanation A., George J., Hayes D.N., Zeisel S., Shores C. (2007). Cancer cachexia syndrome in head and neck cancer patients: Part I. Diagnosis, impact on quality of life and survival, and treatment. Head Neck.

[5] Lango M.N. (2009). Multimodal Treatment for Head and Neck Cancer. Surg. Clin. N. Am..

[6] Almada-Correia I., Neves P.M., Mäkitie A., Ravasco P. (2019). Body Composition Evaluation in Head and Neck Cancer Patients: A Review. Front. Oncol..

[7] Silver H.J., Dietrich M.S., Murphy B.A. (2007). Changes in body mass, energy balance, physical function, and inflammatory state in patients with locally advanced head and neck cancer treated with concurrent chemoradiation after low-dose induction chemotherapy. Head Neck.

[8] Dechaphunkul T., Martin L., Alberda C., Olson K., Baracos V., Gramlich L. (2013). Malnutrition assessment in patients with cancers of the head and neck: A call to action and consensus. Crit. Rev. Oncol. Hematol..

[9] Fouladiun M., Körner U., Bosaeus I., Daneryd P., Hyltander A., Lundholm K.G. (2005). Body composition and time course changes in regional distribution of fat and lean tissue in unselected cancer patients on palliative care—Correlations with food intake, metabolism, exercise capacity, and hormones. Cancer.

[10] Couch M.E., Dittus K., Toth M.J., Willis M.S., Guttridge D.C., George J.R., Chang E.Y., Gourin C.G., Der-Torossian H. (2015). Cancer cachexia update in head and neck cancer: Pathophysiology and treatment. Head Neck.

[11] Pring E.T., Malietzis G., Kennedy R.H., Athanasiou T., Jenkins J.T. (2018). Cancer cachexia and myopenia—Update on management strategies and the direction of future research for optimizing body composition in cancer—A narrative review. Cancer Treat. Rev..

[12] Wendrich A.W., Swartz J.E., Bril S.I., Wegner I., de Graeff A., Smid E.J., de Bree R., Pothen A.J. (2017). Low skeletal muscle mass is a predictive factor for chemotherapy dose-limiting toxicity in patients with locally advanced head and neck cancer. Oral Oncol..

[13] Jackson W., Alexander N., Schipper M., Fig L., Feng F., Jolly S. (2014). Characterization of changes in total body composition for patients with head and neck cancer undergoing chemoradiotherapy using dual-energy X-ray absorptiometry. Head Neck.

[14] Rd H.J., Dijkstra P.U., Vissink A., Langendijk J.A., Van Der Laan B.F., Pruim J., Roodenburg J.L.N. (2011). Changes in nutritional status and dietary intake during and after head and neck cancer treatment. Head Neck.

[15] Lønbro S., Dalgas U., Primdahl H., Johansen J., Nielsen J.L., Overgaard J., Overgaard K. (2013). Lean body mass and muscle function in head and neck cancer patients and healthy individuals—Results from the DAHANCA 25 study. Acta Oncol..

[16] Lonkvist C.K., Vinther A., Zerahn B., Rosenbom E., Deshmukh A., Hojman P., Gehl J. (2017). Progressive resistance training in head and neck cancer patients undergoing concomitant chemoradiotherapy. Laryngoscope.

[17] Ghadjar P., Hayoz S., Zimmermann F., Bodis S., Kaul D., Badakhshi H., Bernier J., Studer G., Plasswilm L., Budach V. (2015). Impact of weight loss on survival after chemoradiation for locally advanced head and neck cancer: Secondary results of a randomized phase III trial (SAKK 10/94). Radiat. Oncol..

[18] Gorenc M., Kozjek N.R., Strojan P. (2015). Malnutrition and cachexia in patients with head and neck cancer treated with (chemo)radiotherapy. Rep. Pract. Oncol. Radiother..

[19] Wang C.-H., Wang H.-M., Pang Y.-P., Yeh K.-Y. (2012). Early nutritional support in non-metastatic stage IV oral cavity cancer patients undergoing adjuvant concurrent chemoradiotherapy: Analysis of treatment tolerance and outcome in an area endemic for betel quid chewing. Support. Care Cancer.

[20] Arends J., Baracos V., Bertz H., Bozzetti F., Calder P., Deutz N., Erickson N., Laviano A., Lisanti M., Lobo D. (2017). ESPEN expert group recommendations for action against cancer-related malnutrition. Clin. Nutr..

[21] Bøje C.R., Dalton S.O., Primdahl H., Kristensen C.A., Andersen E., Johansen J., Andersen L.J., Overgaard J. (2014). Evaluation of comorbidity in 9388 head and neck cancer patients: A national cohort study from the DAHANCA database. Radiother. Oncol..

[22] Bauer J., Capra S., Ferguson M. (2002). Use of the scored Patient-Generated Subjective Global Assessment (PG-SGA) as a nutrition assessment tool in patients with cancer. Eur. J. Clin. Nutr..

[23] Hangartner T.N., Warner S., Braillon P., Jankowski L., Shepherd J. (2013). The Official Positions of the International Society for Clinical Densitometry: Acquisition of Dual-Energy X-Ray Absorptiometry Body Composition and Considerations Regarding Analysis and Repeatability of Measures. J. Clin. Densitom..

[24] Ng K., Leung S.F., Johnson P.J., Woo J. (2004). Nutritional Consequences of Radiotherapy in Nasopharynx Cancer Patients. Nutr. Cancer.

[25] Grossberg A., Chamchod S., Fuller C.D., Mohamed A., Heukelom J., Eichelberger H., Kantor M.E., Hutcheson K., Gunn G.B., Garden A. (2016). Association of Body Composition with Survival and Locoregional Control of Radiotherapy-Treated Head and Neck Squamous Cell Carcinoma. JAMA Oncol..

[26] Conte E., Bresciani E., Rizzi L., Cappellari O., De Luca A., Torsello A., Liantonio A. (2020). Cisplatin-Induced Skeletal Muscle Dysfunction: Mechanisms and Counteracting Therapeutic Strategies. Int. J. Mol. Sci..

[27] Pin F., Barreto R., Couch M.E., Bonetto A., O’Connell T.M. (2019). Cachexia induced by cancer and chemotherapy yield distinct perturbations to energy metabolism. J. Cachex Sarcopenia Muscle.

[28] Zhang Y., Pan X., Sun Y., Geng Y.-J., Yu X.-Y., Li Y. (2018). The Molecular Mechanisms and Prevention Principles of Muscle Atrophy in Aging. Adv. Exp. Med. Biol..

[29] Sin T.K., Zhang G., Zhang Z., Zhu J.Z., Zuo Y., Frost J.A., Li M., Li Y.-P. (2021). Cancer-Induced Muscle Wasting Requires p38β MAPK Activation of p300. Cancer Res..

[30] Malavaki C.J., Sakkas G., Mitrou G.I., Kalyva A., Stefanidis I., Myburgh K., Karatzaferi C. (2015). Skeletal muscle atrophy: Disease-induced mechanisms may mask disuse atrophy. J. Muscle Res. Cell Motil..

[31] Gould D.W., Lahart I., Carmichael A.R., Koutedakis Y., Metsios G.S. (2013). Cancer cachexia prevention via physical exercise: Molecular mechanisms. J. Cachex Sarcopenia Muscle.

[32] Darabseh M.Z., Maden-Wilkinson T.M., Welbourne G., Wüst R.C.I., Ahmed N., Aushah H., Selfe J., Morse C.I., Degens H. (2021). Fourteen days of smoking cessation improves muscle fatigue resistance and reverses markers of systemic inflammation. Sci. Rep..

[33] Li Y.-C., Cheng A.-J., Lee L.-Y., Huang Y.-C., Chang J.T.-C. (2019). Multifaceted Mechanisms of Areca Nuts in Oral Carcinogenesis: The Molecular Pathology from Precancerous Condition to Malignant Transformation. J. Cancer.

[34] Simon L., Jolley S.E., Molina P.E. (2017). Alcoholic Myopathy: Pathophysiologic Mechanisms and Clinical Implications. Alcohol Res. Curr. Rev..

[35] Duan K., Gao X., Zhu D. (2021). The clinical relevance and mechanism of skeletal muscle wasting. Clin. Nutr..

[36] Bozzetti F. (2020). Chemotherapy-Induced Sarcopenia. Curr. Treat. Options Oncol..

[37] Garcia J.M., Scherer T., Chen J.-A., Guillory B., Nassif A., Papusha V., Smiechowska J., Asnicar M., Buettner C., Smith R.G. (2013). Inhibition of Cisplatin-Induced Lipid Catabolism and Weight Loss by Ghrelin in Male Mice. Endocrinology.

[38] Miyawaki E., Naito T., Nakashima K., Miyawaki T., Mamesaya N., Kawamura T., Shota K., Omori H., Wakuda K., Ono A. (2020). Management of anorexia prevents skeletal muscle wasting during cisplatin-based chemotherapy for thoracic malignancies. JCSM Clin. Rep..

[39] Nakano J., Ishii S., Fukushima T., Natsuzako A., Sakamoto F., Natsuzako A., Sakamoto J., Okitaet M. (2017). Factors affecting muscle strength in cancer patients receiving chemotherapy. J. Nov. Physiother. Rehabil..

[40] Willemsen A.C.H., Degens J.H.R.J., Baijens L.W.J., Dingemans A.-M.C., Hoeben A., Hoebers F.J.P., De Ruysscher D.K.M., Schols A.M.W.J. (2020). Early Loss of Fat Mass During Chemoradiotherapy Predicts Overall Survival in Locally Advanced Squamous Cell Carcinoma of the Lung, but Not in Locally Advanced Squamous Cell Carcinoma of the Head and Neck. Front. Nutr..

[41] Powrózek T., Brzozowska A., Mazurek M., Prendecka M., Homa-Mlak I., Mlak R., Małecka-Massalska T. (2021). AA genotype of PLIN1 13041A>G as an unfavourable predictive factor of malnutrition associated with fat mass loss in locally advanced head and neck cancer male patients treated with radiotherapy. Support. Care Cancer.

[42] Donzelli S., Farneti A., Marucci L., Ganci F., Sacconi A., Strano S., Sanguineti G., Blandino G. (2020). Non-coding RNAs as Putative Biomarkers of Cancer-Associated Cachexia. Front. Cell Dev. Biol..

[43] Landrier J.-F., Derghal A., Mounien L. (2019). MicroRNAs in Obesity and Related Metabolic Disorders. Cells.

[44] Byerley L.O., Lee S.H., Redmann S., Culberson C., Clemens M., Lively M.O. (2010). Evidence for a Novel Serum Factor Distinct from Zinc Alpha-2 Glycoprotein That Promotes Body Fat Loss Early in the Development of Cachexia. Nutr. Cancer.

[45] Nazari V., Pashaki A.S., Hasanzadeh E. (2021). The reliable predictors of severe weight loss during the radiotherapy of Head and Neck Cancer. Cancer Treat. Res. Commun..

[46] Willemsen A.C., Hoeben A., Lalisang R.I., Van Helvoort A., Wesseling F.W., Hoebers F., Baijens L.W., Schols A.M. (2020). Disease-induced and treatment-induced alterations in body composition in locally advanced head and neck squamous cell carcinoma. J. Cachex Sarcopenia Muscle.

[47] Ehrsson Y.T., Langius-Eklöf A., Laurell G. (2012). Nutritional surveillance and weight loss in head and neck cancer patients. Support. Care Cancer.

[48] Lønbro S., Petersen G.B., Andersen J.R., Johansen J. (2016). Prediction of critical weight loss during radiation treatment in head and neck cancer patients is dependent on BMI. Support. Care Cancer.

[49] Mangar S., Slevin N., Mais K., Sykes A. (2006). Evaluating predictive factors for determining enteral nutrition in patients receiving radical radiotherapy for head and neck cancer: A retrospective review. Radiother. Oncol..

[50] Mifflin M.D., Jeor S.T.S., Hill L.A., Scott B.J., Daugherty S.A., Koh Y.O. (1990). A new predictive equation for resting energy expenditure in healthy individuals. Am. J. Clin. Nutr..

[51] De Carvalho T.M.R., Marin D.M., Da Silva C.A., De Souza A.L., Talamoni M., Lima C.S.P., Alegre S.M. (2015). Evaluation of patients with head and neck cancer performing standard treatment in relation to body composition, resting metabolic rate, and inflammatory cytokines. Head Neck.

[52] Di Monaco M., Vallero F., Di Monaco R., Mautino F., Cavanna A. (2003). Biochemical Markers of Nutrition and Bone Mineral Density in the Elderly. Gerontology.

[53] Cavanna A., Mautino F., Di Monaco M., Vallero F., Di Monaco R. (2004). Total lymphocyte count and femoral bone mineral density in postmenopausal women. J. Bone Miner. Metab..

[54] Valderrábano R.J., Lui L.-Y., Lee J., Cummings S.R., Orwoll E.S., Hoffman A.R., Wu J.Y., Osteoporotic Fractures in Men (MrOS) Study Research Group (2017). Bone Density Loss Is Associated With Blood Cell Counts. J. Bone Miner. Res..

[55] Ye X., Jiang H., Wang Y., Ji Y., Jiang X. (2020). A correlative studies between osteoporosis and blood cell composition: Implications for auxiliary diagnosis of osteoporosis. Medicine.

[56] Chusyd D.E., Wang D., Huffman D.M., Nagy T.R. (2016). Relationships between Rodent White Adipose Fat Pads and Human White Adipose Fat Depots. Front. Nutr..

[57] Rakotoarivelo V., Lacraz G., Mayhue M., Brown C., Rottembourg D., Fradette J., Ilangumaran S., Menendez A., Langlois M.-F., Ramanathan S. (2018). Inflammatory Cytokine Profiles in Visceral and Subcutaneous Adipose Tissues of Obese Patients Undergoing Bariatric Surgery Reveal Lack of Correlation with Obesity or Diabetes. EBioMedicine.

[58] Alemán H., Esparza J., Ramirez F.A., Astiazaran H., Payette H. (2011). Longitudinal evidence on the association between interleukin-6 and C-reactive protein with the loss of total appendicular skeletal muscle in free-living older men and women. Age Ageing.

[59] Lee S.W., Youm Y., Lee W.J., Choi W., Chu S.H., Park Y.-R., Kim H.C. (2015). Appendicular Skeletal Muscle Mass and Insulin Resistance in an Elderly Korean Population: The Korean Social Life, Health and Aging Project-Health Examination Cohort. Diabetes Metab. J..

[60] Lin Y.-L., Wang C.-H., Lai Y.-H., Kuo C.-H., Syu R.-J., Hsu B.-G. (2018). Negative correlation between leptin serum levels and sarcopenia in hemodialysis patients. Int. J. Clin. Exp. Pathol..

[61] Möller-Loswick A.-C., Bennegård K., Lundholm K. (1991). The forearm and leg perfusion techniques in man do not give the same metabolic information. Clin. Physiol..

[62] Going S.B., Massett M., Hall M.C., A Bare L., A Root P., Williams D.P., Lohman T.G. (1993). Detection of small changes in body composition by dual-energy x-ray absorptiometry. Am. J. Clin. Nutr..

