# Supplementary material for: Concurrent Chemoradiotherapy Induces Body Composition Changes in Locally Advanced Head and Neck Squamous Cell Carcinoma: Comparison between Oral Cavity and Non-Oral Cavity Cancer
Source: Nutrients. 2021 Aug 26;13(9):2969. doi: 10.3390/nu13092969 (PMC8472371; doi:10.3390/nu13092969)
Supplement: Supplementary file 1 [file nutrients-13-02969-s001.zip › nutrients-1344691-supplementary.pdf]

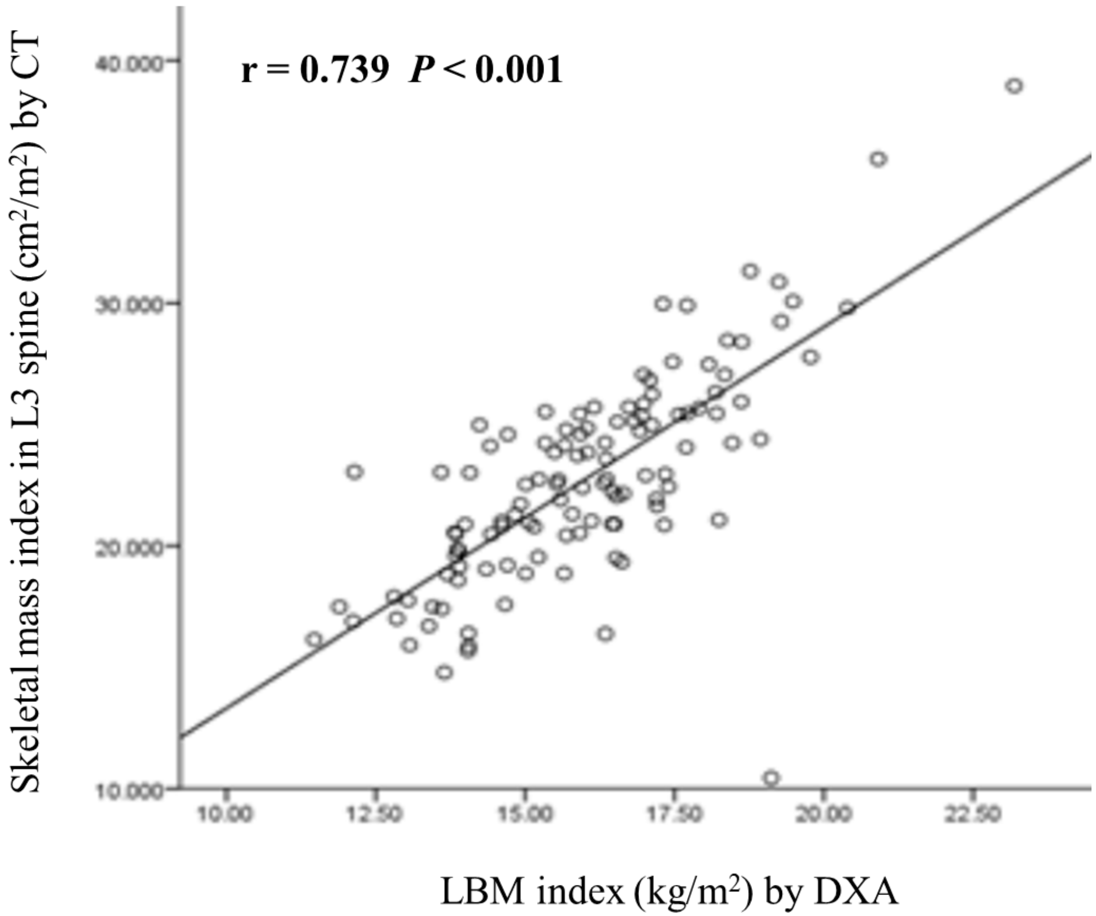

Figure S1. Significant correlation between LBM index assessed by DXA and skeletal muscle index assessed by CT scan of abdomen.

Table S1. The associations between age, BMI, clinicopathologic variables, treatment-related factors and nutritional/inflammatory markers in 127 LAHNSCC patients before CCRT

	Age (years)		BMI (kg/m^2)	
	**Oral cavity with adjuvant CCRT**		**Non-oral cavity with primary CCRT**	
**Included patient number**	**69**		**58**	
**Variables, expressed as correlation coefficient or mean±standard deviation**				
Clinicopathologic factors		*P*-value*		*P*-value*
Age (years)	---	---	0.005	0.972
Sex (male *vs* female)	53.2 ± 5.8 *vs* 58.0 ± 0.0	0.575	20.4 ± 6.6 *vs* 22.9 ± 3.7	0.122
TNM stage (III *vs* IVA *vs* IVB)	51.5 ± 6.4 *vs* 53.0 ± 9.5 vs 54.4 ± 3.9	0.790	23.2 ± 1.5 *vs* 23.4 ± 4.1 vs 21.0 ± 3.6	0.120
T status (T1-2 *vs* T3-4)	57.5 ± 7.9 *vs* 52.7 ± 8.5	0.131	24.1 ± 4.0 *vs* 22.4 ± 3.7	0.067
N status (N0-1 *vs* N2-3)	54.1 ± 7.7 *vs* 52.5 ± 9.0	0.442	22.9 ± 3.2 *vs* 22.7 ± 4.1	0.899
Histologic grade (1 *vs* 2 *vs* 3)	51.6 ± 6.5 *vs* 53.6 ± 9.1 vs 52.4 ± 6.3	0.781	20.9 ± 3.6 *vs* 22.8 ± 4.4 vs 23.0 ± 3.1	0.422
Smoking (no *vs* yes)	55.8 ± 8.4 *vs* 53.0 ± 8.5	0.459	22.9 ± 5.9 *vs* 22.7 ± 3.7	0.928
Alcohol (no *vs* yes)	54.6 ± 7.6 *vs* 52.8 ± 8.7	0.445	22.3 ± 4.6 *vs* 22.9 ± 3.7	0.594
Betel nut (no *vs* yes)	54.9 ± 8.7 *vs* 52.7 ± 8.4	0.380	22.1 ± 4.0 *vs* 23.5 ± 3.8	0.174
ECOG performance status (0 : 1 : 2)	50.0 ± 1.4 *vs* 53.2 ± 9.5 vs 51.0 ± 13.2	0.810	24.8 ± 2.9 *vs* 22.5 ± 4.0 vs 22.3 ± 3.1	0.302
HN-CCI (0 *vs*1 *vs* 2 *vs* ≥3)	50.2 ± 8.1 *vs* 52.0 ± 7.4 vs 53.2 ± 4.4	0.675	22.9 ± 4.4 *vs* 22.1 ± 4.1 vs 23.1 ± 3.3	0.965
Trachesotomy (no *vs* yes)	55.8 ± 8.0 *vs* 52.0 ± 8.4	0.077	23.4 ± 6.6 *vs* 21.8 ± 4.8	0.075
Mean daily calorie intake during CCRT (kcal/kg/day)	0.109	0.374	-0.550	< 0.001*

Treatment-associated factors				
CCRT Regimen				
RT dose (Gy)	0.104	0.396	0.176	0.187
RT fractions	0.118	0.334	0.037	0.781
RT duration (days)	0.067	0.585	0.111	0.407
Cisplatin dose (mg/m^2)	-0.011	0.926	-0.045	0.738
CCRT-induced grade 3/4 toxicity				
Dermatitis (no *vs* yes)	53.3 ± 8.6 *vs* 52.6 ± 2.9	0.907	22.7 ± 3.9 *vs* 24.0 ± 5.4	0.609
Pharyngitis (no *vs* yes)	53.2 ± 8.4 *vs* 52.8 ± 9.7	0.907	22.9 ± 3.8 *vs* 22.1 ± 4.4	0.573
Infection (no *vs* yes)	52.3 ± 8.7 *vs* 52.9 ± 7.4	0.895	23.2 ± 3.7 *vs* 21.8 ± 4.4	0.192
Mucositis (no *vs* yes)	54.0 ± 9.1 *vs* 51.8 ± 5.8	0.190	23.0 ± 4.0 *vs* 22.1 ± 3.7	0.488
Emesis (no *vs* yes)	53.5 ± 8.7 *vs* 51.0 ± 5.6	0.442	22.9 ± 3.9 *vs* 21.5 ± 3.7	0.498
Anemia (no *vs* yes)	53.1 ± 8.5 *vs* 54.6 ± 9.5	0.711	22.7 ± 3.7 *vs* 23.4 ± 5.8	0.664
Neutropenia (no *vs* yes)	52.9 ± 8.6 *vs* 53.8 ± 8.3	0.662	22.8 ± 3.7 *vs* 22.7 ± 4.3	0.928
Thrombocytopenia (no *vs* yes)	53.2 ± 9.5 *vs* 52.5 ± 9.9	0.860	22.6 ± 3.9 *vs* 24.2 ± 3.7	0.300
Nutritional/inflammatory markers before CCRT				
PG-SGA (well *vs* moderate *vs* severe)	52.4 ± 10.0 *vs* 54.7 ± 8.4 vs 50.7 ± 7.2	0.242	21.7 ± 2.3 *vs* 22.8 ± 3.8 vs 21.2 ± 4.1	0.068
BMI (kg/m^2)	0.009	0.944	--	--
BW (kg)	-0.116	0.344	0.914	< 0.001*
Hb (g/dL)	-0.199	0.102	0.239	0.070
WBC (x10^3 cells/mm^3)	-0.125	0.308	0.081	0.544
Platelet (x10^3 /mm^3)	-0.058	0.637	0.208	0.117

TLC (x10^3 cells/mm^3)	-0.034	0.782	0.295	0.025
Albumin (g/dL)	-0.201	0.069	0.497	< 0.001*
CRP (mg/dL)	0.200	0.100	0.188	0.166
Resting metabolic Rate (Kcal/day)	-0.438	< 0.001*	0.732	<0.001*

*The *P*-value was determined by Pearson correlation analysis (for age, mean daily calorie, RT dose, RT fraction, RT days, cisplatin dose, BMI, BW, Hb, WBC, platelet, TLC, albumin, CRP and resting metabolic rate), ANOVA using Bonferroni adjustments (for histologic grade, ECOG performance status, HN-CCI and PG-SGA), nonparametric statistics with Kruskal–Wallis H test (for TNM stage) for multiple comparisons, independent *t* test (for sex, T status, N status, smoking, alcohol, betel nut, tracheostomy, and all CCRT-induced grade 3/4 toxicities). $P < 0.05$ represents statistical significance.

** It was calculated by Harris–Benedict equation [1].

Abbreviations: LAHNSCC , locally advanced head and neck squamous cell carcinoma; CCRT, concurrent chemoradiotherapy; HN-CCI, head and neck-Charlson Comorbidity Index; ECOG, Eastern Cooperative Oncology Group; PG-SGA, patient generated subjective global assessment; RT, radiotherapy; BW, body weight; BMI, body mass index; Hb, hemoglobin; WBC, white blood cell; TLC, total lymphocyte count; CRP, C-reactive protein.

Reference:

1. Mifflin MD, St Jeor ST, Hill LA, Scott BJ, Daugherty SA, Koh YO (1990). A new predictive equation for resting energy expenditure in healthy individuals. *The American Journal of Clinical Nutrition*. **51** (2): 241–7.
